# Return to self-reported physical activity level after an event of acute low back pain

**DOI:** 10.1371/journal.pone.0219556

**Published:** 2019-07-10

**Authors:** Daniel Pozzobon, Leandro A. Calazans Nogueira, Paulo H. Ferreira, Daniel Steffens, Paula R. Beckenkamp, Fiona M. Blyth, Manuela L. Ferreira

**Affiliations:** 1 Institute of Bone and Joint Research, the Kolling Institute, Sydney Medical School, University of Sydney, Sydney, NSW, Australia; 2 Rehabilitation Science Post-graduate Program, Augusto Motta University Centre (UNISUAM), Rio de Janeiro, RJ, Brazil; 3 Physiotherapy Department at Federal Institute of Rio de Janeiro (IFRJ), RJ, Brazil; 4 The University of Sydney, Musculoskeletal Health, Faculty of Health Sciences, Discipline of Physiotherapy, Sydney, NSW, Australia; 5 Surgical Outcomes Research Centre (SOuRCe), Royal Prince Alfred Hospital (RPAH), Sydney, NSW, Australia; 6 Concord Clinical School, University of Sydney, Sydney, NSW, Australia; Monash University, AUSTRALIA

## Abstract

**Background:**

Regular physical activity participation is known to promote better mobility and coordination. Although previous research has established that lack of physical activity participation may increase the risk of developing low back pain, the role of physical activity as a predictor of recovery among symptomatic individuals remains unclear.

**Objective:**

To evaluate whether: (i) the level of physical activity participation before an acute episode of low back pain predicts recovery within 12 months following the acute pain episode (i.e. index episode); and (ii) participants return to their pre-pain level of physical activity participation 12 months from the index episode.

**Design:**

This study used longitudinal data from the TRIGGERS case-crossover study.

**Setting:**

This study was conducted through over-the-phone interviews to participants that presented to 300 primary care clinics with a new episode of acute low back pain.

**Participants:**

This study included 999 consecutive patients, aged 18 years or older. Follow-up assessment was completed at 12 months following the index episode.

**Methods:**

This is a cohort study where consecutive patients, aged 18 years or older, that presented with a new episode of acute low back pain were recruited between October 2011 and November 2012.

**Main outcome measurements:**

Self-reported level of physical activity participation.

**Results:**

A total of 830 participants completed the study. When comparing participants who reported pain at 12 months follow-up with those without pain, all participants reported similar levels of physical activity participation one week before (p = 0.449), one week after (p = 0.812) and 12 months after the index episode (p = 0.233). The level of physical activity participation before the index episode was not a reliable predictor of presence of pain at either 3 or 12 months follow-up (OR 0.99; 95% CI 0.993 to 1.003; p = 0.523 and OR 1; 95% CI 0.992 to 1.008; p = 0.923, respectively).

**Conclusion:**

Physical activity participation did not predict recovery from the pain episode. Also all participants returned to their pre-pain level of physical activity participation after 12 months.

## Introduction

Low back pain is a common condition, with a lifetime prevalence of approximately 80% [1, 2]. Given its high prevalence, the costs associated with this condition are considerable. In the United Kingdom, the total annual health-care expenditure related to low back pain is estimated at ₤ 11 billion, and in the United States the annual cost with treatment for low back pain is estimated to be US$ 90 billion [3]. A recent report shows that over 3.2 million Australian reported chronic pain from either arthritis or back pain and the majority of these individuals (56%) also reported activity limitations due to the pain [4]. The prevalence of chronic pain is expected to affect over 5.2 million Australians by 2050 with over 2.9 million individuals experiencing activity limitation due to pain [4].

Regular physical activity participation is known to promote better mobility and coordination, overall fitness, and muscle power and rapid strength [5] as well as decreasing mortality risk [6] and cardiovascular disease risk [7]. Physical activity participation has been identified as a potential protective factor for a number of musculoskeletal conditions, including low back pain [8] [9], however it is still unknown if physical activity participation before an episode will impact the course of this condition. Some domains of physical activity, such as frequency (e.g. when too low) and intensity (e.g. when too high), are known as risk factors for the onset of low back pain [10, 11]. This study uses data on physical activity intensity and duration to calculate the metabolic demand and total metabolic expenditure minutes (MET min/week) of the reported physical activities. Although previous research has established that both lack of regular physical activity participation and engagement in strenuous physical activity may increase the risk of developing low back pain [12], the role of physical activity participation as a predictor of recovery among symptomatic individuals remains unclear. Moreover, it is unknown whether individuals who recover from an episode of low back pain ever return to their pre-pain levels of physical activity participation. This would be especially important since the regular practice of physical activity is known to be related to better mobility, muscle power and overall health [5, 9].

Therefore, the aim of this study was to evaluate whether: (i) the self-reported level of physical activity participation before an acute episode (index episode) of low back pain predicts recovery within 12 months following the index pain episode; and (ii) participants return to their pre-pain self-reported level of physical activity participation 12 months from the index episode.

## Methods

### Study design

We hypothesised that participants with higher levels of physical activity (considering duration and intensity of activity) before the index episode would be more likely to recover within the first 12 months. We also hypothesized that those who recovered from their pain episode would be more likely to return to their original (i.e. pre pain) levels of physical activity participation. We used longitudinal data from the TRIGGERS for low back pain Study. The study was approved by the Human Research Ethics Committee of the University of Sydney (protocol number 05-2011/13742). The TRIGGERS Study was a case-crossover study designed to investigate a number of transient physical and psychosocial risk factors for an episode of sudden-onset, acute low back pain. Follow-up assessment was completed at 3 and 12 months following the index episode of pain, through telephone interviews. All participants were assessed by the same team of researchers, following the same standardised assessment booklet. Detailed information has been published elsewhere [13].

### Participants

The study included 999 consecutive patients, aged 18 years or older, that presented to 300 primary care clinics (general practitioners, physical therapists, and chiropractors) with a new episode of acute low back pain (i.e. index episode). All participants gave written informed consent for participation. Participants were referred by the clinics and recruited between October 2011 and November 2012 and interviewed to assess the inclusion/exclusion criteria previously described in the study protocol [14]. A new episode of acute low back pain was defined as a primary complaint of pain between the 12th rib and the buttock crease, with or without leg pain, causing the patient to seek health care, and preceded by a period of at least 1 month without back pain [15]. Patients needed to present to primary care within 7 days from index episode and report pain of at least moderate intensity in the first 24 hours (measured using item 7 of the Short Form 36 questionnaire: “How much bodily pain have you had during the past 4 weeks? None; Very mild; Mild; Moderate; Severe or Very severe.”) [16]. Participants with known or suspected serious spinal pathology (metastatic, inflammatory or infective diseases of the spine) were excluded.

### Outcome measures

At study entry, a telephone-based interviewed was conducted within 7 days following referral to the study. At baseline, participants were asked about their age, gender, height, weight, medication taken for the pain, employment status, type of work, manual tasks performed (e.g. lifting heavy loads, awkward positioning, handling objects far from the body), a recent slit, trip or fall, pain intensity, feelings of depression or anxiety, alcohol consumption, as well as engagement in moderate or vigorous physical activity and sexual activity. The date and time of pain onset was also identified with the assistance of recommended recall aids, such as a diary and/or smartphone. Participants’ BMI was calculated based on the self-reported height and weight. Participants were also asked to report pain intensity at the moment of the interview using a numeric rating scale (0 to 6) and disability (0 to 5) based on item 7 of the SF-36 questionnaire [17]. The SF-36 is a valid and reliable self-administered tool for measurement of physical and social functioning [17]. The SF-36 correlation coefficient with the International Quality of Life Assessment (IQOLA) for the physical functioning section is 0.85 and 0.42 for the social functioning section, and the internal consistency reliability coefficient for the physical functioning is 0.93 and 0.68 for social functioning.[18]. The overall internal consistency reliability of SF-36 is estimated to be 0.87 [19]. Tension or anxiety and feelings of depression were assessed based on items 13 and 14 of the Orebro Musculoskeletal Pain Questionnaire (“How tense or anxious have you felt in the past week?” and “How much have you been bothered by feeling depressed in the past week?”, respectively) [20].

The self-reported level of physical activity participation was assessed using the Active Australia Survey (AAS) questionnaire [21], where participants estimated the total number of hours they spent engaged in light, moderate, and vigorous physical activity in the week preceding the index episode, and at 1 week and at 12 months follow-up. Participants were contacted again at 3 months and 12 months and were asked if they had recovered from the index episode, and in case of a positive answer, the date when the pain fully resolved was used to calculate the duration of episode. Recovery was defined as being pain-free for at least one month before the follow-up interview [15]. This design has already been successfully used to assess physical activity participation among persons seeking care due to low back pain [22]. Participants were also asked at baseline and follow-up surveys about the presence and intensity of symptoms of depression and anxiety.

### Assessment of frequency and intensity of physical activity

In this study, physical activity was defined as any bodily movement that increases the metabolic demand above the rest state [23]. Participants were asked about the total time spent in physical activity of different intensities (e.g. light, moderate or vigorous) the week before pain onset. Pain onset could have been up to 2 weeks prior to the baseline interview. The AAS questionnaire was developed focusing on the recall period of 1 week and has been shown to present estimates of physical activity level comparable with pedometer steps and accelerometer counts and considers physical activity of light intensity (e.g. walking briskly), moderate intensity (e.g. gentle swimming and recreational tennis) and vigorous intensity (e.g. cycling and competitive tennis) [24]. The AAS was developed based on the International Physical Activity Questionnaire (IPAQ) therefore we used the IPAQ-SF scoring system to calculate the metabolic demand and total metabolic expenditure minutes (MET min/week) following the scoring system used in the short form of the IPAQ (IPAQ-SF) for different levels of intensity of physical activity (i.e. light = 3.3 met/min, moderate = 4 met/min and vigorous = 8 met/min) [25] for each participant at each time point.

### Statistical analysis

Patients were split into two groups according to the presence of pain at the 12-month follow-up assessment; ‘recovered’ if they no longer reported pain and ‘not recovered’ if they reported persistent pain. Baseline demographic variables were summarised as mean (SD) if continuous and frequency (percent) if categorical.

Generalised estimating equations were used to investigate the change in total metabolic equivalent minutes over time. A time-by-group interaction term was included alongside main effects recovery group and time-point (one-week prior to episode, week of episode and 12 months after episode) were to conduct within- and between-group comparisons at each time point as well as across all time points.

Logistic regression models were used to assess the association between levels of physical activity one-week prior to the pain episode and the risk of non-recovery at 12 months.

All models were adjusted for known confounders including age, gender, BMI, pain intensity at index episode and depression or anxiety symptoms assessed based on Orebro Musculoskeletal Pain Questionnaire [8, 20].

Total Metabolic Equivalent minutes per week (MET min/week) was used to quantify physical activity participation prior to index episode and used in the analysis to predict the presence of low back pain at the 12-month follow-up. We used the scoring system from the IPAQ-SF since it has been validated against accelerometer data [25] and it is a well-known way to pool physical activities of different intensities to facilitate the comparison between different activities [26]. Generalised linear models were conducted using total MET min/week as the dependent variable (continuous) considering each time-point (1 week before low back pain episode, week of the low back pain episode and 12 months after the episode) and the presence of low back pain at 3 months and 12 months.

A sensitivity analysis was conducted, repeating the analyses on the 3-month follow-up assessment [27]. All analyses were conducted using IBM SPSS Statistics for Windows, Version 22.0 (Armonk, NY: IBM Corp.) Statistical significance was set as p < 0.05.

## Results

**Fig 1:**
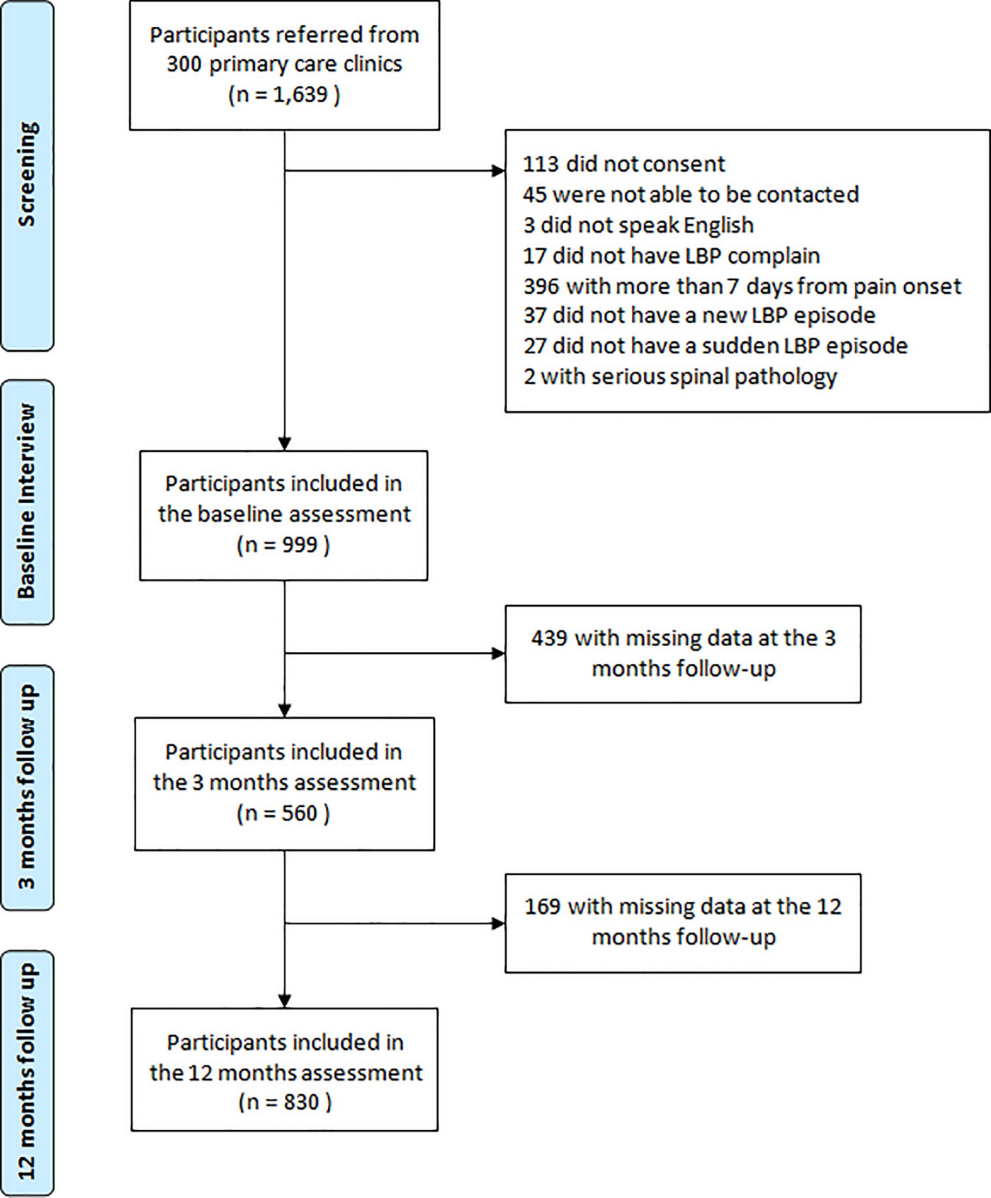
Flowchart of participant’s recruitment and follow-up participation.

Of the 999 participants initially included, 83% had complete follow-up data and were included in our study. The main reasons for not obtaining follow-up data were participants being unavailable to respond interview (n = 139) or declining to participate (n = 28) (Fig 1). The 830 participants presented at baseline with mean low back pain duration of 4.9 (SD 3.5) days, and mean time from pain onset to presentation to primary care of 3.0 (SD 2.1) days and 4.9 (SD 2.7) days from presentation to baseline interview. Participants’ demographic and clinical characteristics are presented in Table 1. At the 12-month follow-up, 661 participants had recovered from the initial episode of low back pain, while 169 remained symptomatic and reported having an average of 1.7 (SD 1.2) self-reported pain intensity (0 to 6 scale) and 1.4 (SD 0.8) self-reported disability (0 to 5 scale). Of the 661 who reported being recovered at 12 months, 597 recovered within the first 3 months post index episode.

**Table 1:** Baseline characteristics of the participants presented as mean (standard deviation), unless otherwise stated.

Characteristics	Mean (SD)		p-value^‡^
	Persistent pain at the 12-month follow-up (N = 169)	Recovered at 12-month follow-up (N = 661)	
Age, years	45.9 (13.8)	45.2 (13.4)	0.30
Gender (female/male)	76 / 94	304 / 356	
Body mass index (kg/m^2^)	26.4 (4.5)	26.4 (5.5)	0.94
Days of episode^α^	5 (2.7)	4.9 (2.7)	0.55
Days to seek care	3.1 (2.0)	2.9 (2.1)	0.07
Days to recovery^β^		50.4 (58.3)	
Pain intensity at event (%)			
Moderate	60 (35.5)	255 (38.7)	**0.02**
Severe	77 (45.6)	330 (50.1)	**0.02**
Very Severe	32 (18.9)	74 (11.2)	**0.02**
Occupation n (%)			
Unemployed	24 (14.0)	118 (17.9)	**0.01**
Clerical and Administrative Worker	16 (9.4)	64 (9.7)	**0.01**
Community and Personal Service Worker	8 (4.7)	34 (5.2)	**0.01**
Labourer	6 (3.5)	19 (2.9)	**0.01**
Machinery Operator and Driver	3 (1.8)	20 (3.0)	**0.01**
Manager	30 (17.5)	102 (15.5)	**0.01**
Professional	67 (39.2)	216 (32.7)	**0.01**
Sales worker	4 (2.3)	36 (5.5)	**0.01**
Technician and Trade Worker	13 (7.6)	51 (7.7)	**0.01**
Physical activity participation level (total MET minutes/week)			
1 week before event	2,034 (2,499)	1,892 (2,452)	0.45
Week of event	1,245 (2,488)	1,281 (2,451)	0.81
12 months after event	1,626 (2,472)	1,851 (2,471)	0.23

^‡^Bold denotes significance at the 0.05 level

α - Time elapsed between the date of pain onset and the date of the baseline interview

β - Time elapsed between the date of pain onset and the date of reported recovery.

### Change in physical activity participation

**Fig 2:**
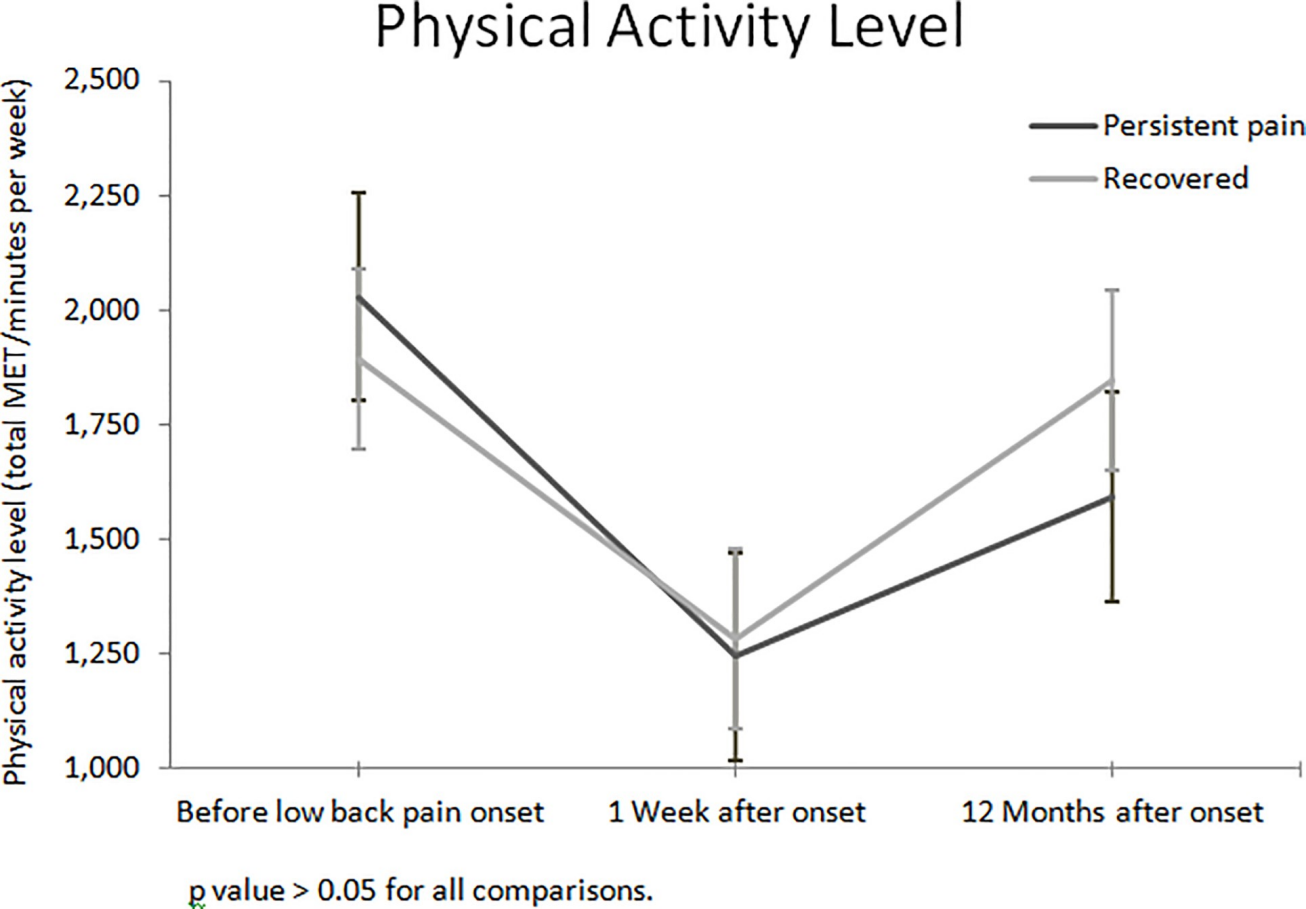
Mean total MET minutes per week at each time-point. p value > 0.05 for all comparisons.

For the physical activity participation analyses, participants were grouped according to their pain status at 12 months. Both groups (i.e. participants with persistent pain and participants who had recovered) reported similar levels of physical activity participation at all time-points assessed: one week before the index episode (p = 0.449), one week after index episode (p = 0.812) and 12 months after the index episode (p = 0.233) (Table 2). Participants reported a statistically significant reduction in the mean MET minutes/week one week after the index episode in both persistent pain (mean METs minutes/week: -788; 95% confidence interval [CI]: -990 to -585; p<0.001) and recovered (METs minutes/week: -611; 95% CI: -723 to -498; p<0.001) groups. At 12 months, both groups reported increases in the levels of physical activity participation similar to pre-pain levels (Fig 2). The generalised linear model confirmed a similar pattern for physical activity participation between groups after adjusting for age, gender, BMI, pain intensity at event and depression, at both 3 and 12 months after the index episode (χ^2^ = 3.622, p = 0.16 and χ^2^ = 2.963, p = 0.23, respectively).

**Table 2:** Difference in physical activity level (total MET minutes per week) before the onset of low back pain within group among multiple time-points.

	Pre-pain physical activity participation	Physical activity participation 1 week after index episode	Change from pre-pain levels of physical activity	Physical activity participation 12 months after index episode	Change from pre-pain levels of physical activity
Persistent pain group (SD)/(95% CI)	2,034 (2,499)	1,245 (2,488)	**-788.3 (-990 to -585); p<0.001**	1,626 (2,472)	-407.3 (-822 to 8.38); p = 0.055
Recovered group (SD)/(95% CI)	1,892 (2,452)	1,281 (2,451)	**-611.1 (-723 to -498); p<0.001**	1,851 (2,471)	-41.1 (-300.6 to 218.3); p = 0.756
Between-group difference (95% CI)	141 (-508.1 to 224.8); p = 0.449	35.6 (-257.1 to 328.3); p = 0.812	-	224.5 (-144.5 to 593.6); p = 0.233	-

Bold denotes significance at the 0.05 level.

The results of our logistic regression models showed that baseline physical activity participation (i.e., before index episode) was not a predictor of recovery at 12 months, after adjusting for age, gender, BMI, pain intensity at baseline and depression or anxiety symptoms (AOR 0.99; 95% CI 0.99 to 1.00; p = 0.523)(Table 3). Similarly, when considering those who had recovered within the first 3 months post index episode, our sensitivity analysis showed that the amount of physical activity before index episode did not predict recovery at 3 months (OR 1; 95% CI 0.99 to 1.00; p = 0.923) (Table 3). The results of unadjusted logistic regression models are presented in Table 4.

**Table 3:** Association between physical activity level (total MET minutes per week) before index episode and the presence of low back pain 3 months and 12 months after the index episode.

Presence of pain*	Physical Activity Level			
	Odds Ratio	95% CI Lower limit	95% CI Upper limit	P value
3 months	1	0.99	1.01	0.92
Age (years)	1	0.99	1.01	0.94
Gender (Male)	0.72	0.49	1.06	0.09
BMI (kg/m^2^)	0.96	0.92	0.99	**0.04**
Pain intensity at index episode	0.91	0.83	0.99	**0.03**
12 months	0.99	0.99	1.00	0.52
Age (years)	1	0.99	1.01	0.42
Gender (Male)	1.11	0.88	1.39	0.37
BMI (kg/m^2^)	1	0.99	1.03	0.41
Pain intensity at index episode	1.06	1.01	1.12	**0.02**

*Both analyses were adjusted for age, gender, BMI, pain intensity at index episode, depression, anxiety and occupation. Bold denotes significance at the 0.05 level.

**Table 4:** Association between physical activity level (total MET minutes per week) before index episode and the presence of low back pain 3 months and 12 months after the index episode (unadjusted analysis).

Presence of pain	Physical Activity Level			
	Odds Ratio	95% CI Lower limit	95% CI Upper limit	P value
3 months	1	1	1	0.77
12 months	1	1	1	0.56

## Discussion

Our results have shown that the self-reported level of physical activity participation before pain onset was not associated with recovery 12 months following the index episode, after adjusting for age, gender, BMI, depression and pain intensity at index episode. In fact, pre pain levels of physical activity were similar for those who developed persistent pain, compared to those who recovered within 12 months from index episode. Similar results were also observed for those who had recovered by 3 months after index episode. Our results also demonstrated that, although there is a sharp reduction in physical activity participation in the first week after the index episode, most people reached similar pre-pain levels of physical activity 12 months after index episode.

Our study used a large inception cohort where participants were included near or soon after the onset of symptoms. Also, our sample had a low exclusion rate (11.9%), mainly due to low pain intensity, suggesting ours was a representative sample of the population seeking medical attention for an acute episode of low back pain. We acknowledge however, that this was a physically active cohort, and only 17.4% of participants were considered inactive before pain onset, according to IPAQ-SF classification score [28]. This suggests our cohort was, on average, more active than the general population, with a weekly mean of 449 (SD 544) minutes of physical activity participation (over 60 minutes per day) versus the mean 39 minutes per day observed in the general population [29]. Typically, our cohort was also relatively young (45.3 years), most of them employed as clerical and administrative workers, community and personal service workers, managers or sales workers (84%). It is possible that our study population was more health literate and, therefore, more aware of the benefits of regular physical activity participation.

Our findings agree with the results of a systematic review assessing the relationship between physical activity and pain and disability including measures of recovery and reoccurrence of low back pain [30]. The authors included 12 studies (seven cohort and five cross-sectional) in their analyses (n = 3,979) to assess the role of habitual physical activity engagement on disability scores or pain levels and found no support on the literature on the association of physical activity and non-specific low back pain outcome measures. These findings support our results of no significant association between physical activity levels and the outcomes, recovery or reoccurrence of low back pain. Our study adds to the body of evidence relating the self-reported level of physical activity engagement to low back pain by following a sample of enough size and with long term follow-up data collections to assess the predictive value of the self-reported level of physical activity engagement for recovery after an acute episode of low back pain.

Although physical activity participation was not found to be associated with recovery from a low back pain episode, when taking into consideration the benefits linked to regular physical activity participation it is likely that avoiding bed rest and remaining as physically active as possible should continue to be recommended as the management of low back pain. Also, based on our results it is safe to say that engagement in physical activities did not cause further harm, supporting the current recommendation that participants should avoid bed rest and try to resume their activities routines as soon as possible [31].

### Study limitations

Our study presents some limitations that need to be considered when interpreting the results. For instance, 17% of participants were lost to follow-up due to unavailability to respond or declining to participate in the study. We also acknowledge that physical activity participation was ascertained via questionnaire. There is evidence suggesting that self-reported measures of physical activity participation result in overestimation of participation when compared to accelerometer-based data, especially in cohorts with moderate to high intensity physical active routines [32]. The Active Australia questionnaire also relies on recall, as participants were asked about their level of physical activity on the week before the interview; however, short recall periods of up to 2 weeks are reported as less susceptible to recall bias [21]. This could be a potential source of bias since the perception of pain could lead the participants to change their perceptions of how much physical activity they performed on the week prior to the pain, however, despite the maximum recall time possible in our study being of up to 3 weeks, the actual mean recall time was of only 5 days [13]. Due to the high variability of the physical activity measurement and the recall bias, these could have biased the results of the assessed associations towards the null.

## Conclusion

Our study has shown that, regardless of the self-reported level of physical activity participation before an episode of acute low back pain, and whether or not they have recovered from pain at 12 months, participants with low back pain tend to return to the pre-pain self-reported level of physical activity. Moreover, physical activity participation before pain onset is not a significant predictor of recovery among people with an acute onset of low back pain.

## Abbreviations

MET: Metabolic expenditure
SF-36: Short Form 36 questionnaire
IPAQ-SF: International Physical Activity Questionnaire–Short Form
SD: Standard deviation
BMI: Body Mass Index
CI: Confidence Interval
